# Community participation and stakeholder engagement in determining health service coverage: A systematic review and framework synthesis to assess effectiveness

**DOI:** 10.7189/jogh.13.04034

**Published:** 2023-05-12

**Authors:** Megan Arthur, Ria Saha, Anuj Kapilashrami

**Affiliations:** 1 Global Health Policy Unit, School of Social and Political Science, University of Edinburgh, Edinburgh, UK; 2 The Food Foundation, London, UK; 3 School of Health and Social Care, University of Essex, Colchester, UK

## Abstract

**Background:**

Community and stakeholder involvement in decision-making to determine publicly-funded health services and interventions is advocated to fulfil citizens’ rights and improve health outcomes. The inclusion of public actors, particularly disadvantaged populations, in priority setting for universal health coverage (UHC) is also enshrined in guidance from the World Health Organization (WHO). However, challenges remain in operationalising this policy aim and ensuring that these approaches are effective and equitable. This study aimed to synthesise published evidence on the role of community and stakeholder participation in determining health service coverage.

**Methods:**

A systematic review was conducted, searching the Web of Science, Ovid Global Health, and PubMed Central databases from 2000 onwards, including all study types. A framework synthesis approach was used for charting and synthesising data on mechanisms, effectiveness (equity, depth, and stage), and barriers and facilitators for engagement.

**Results:**

Twenty-seven relevant studies were identified that involve community actors and other stakeholders in priority setting and decision-making processes for defining health benefit packages and UHC, health technology assessment, and pharmaceutical coverage. Mechanisms of engagement include a wide variety of consultation approaches; participation in decision-making committees, advisory councils, and local planning meetings; and appeals mechanisms. Participation occurs primarily at Data and Dialogue stages of decision-making processes, and we found limited depth of engagement among identified cases. Limited consideration of equity was observed in planning and reporting on community involvement in priority setting. A number of challenges are identified in the literature, which we typologise as institutional, procedural, technical, and structural / normative barriers to meaningful participation.

**Conclusions:**

This systematic review identifies key gaps and opportunities in the literature and practice related to effective and equitable community and stakeholder participation in determining health service coverage. It offers essential considerations for planning and executing inclusive approaches to priority setting for publicly-funded health services and interventions and defining health benefit packages for UHC.

Decision-makers around the world face the challenge of setting priorities and determining the publicly-funded health services and interventions available to a population. Increasingly, calls for greater involvement of community and other stakeholders in these processes are being made by academics, policy actors, and practitioners globally, including in the global policy agenda for social participation for universal health coverage (UHC) [1]. Framed as central to primary health care architecture [2] and enshrined as a core principle of rights-based approaches to health [3], community participation in decision-making for health services has gained currency over the past decade.

Understandings of community and stakeholder participation (CSP) are contested however, in part due to ambiguity and different normative understandings of “community” and “participation”. In their framework for community engagement, the World Health Organization (WHO) defines community participation as “a process of developing relationships that enable stakeholders to work together to address health-related issues and promote well-being to achieve positive health impact and outcomes” [4]. Despite mounting interest, challenges remain in operationalising CSP and ensuring that engagement is meaningful and effective. Studies examining community participation in health systems and health care planning are largely concentrated on downstream service delivery and monitoring of interventions, rather than upstream governance and priority setting for defining a core package of publicly-funded health services, reflecting an important gap in the literature. Reviews in the literature on priority setting in health and social policy have found a dearth of evidence and evaluation of CSP [5-7], however to the best of our knowledge no study to date has yet focused on priority setting for health service coverage in particular.

In our conceptualisation of health service coverage, we include priority setting and decision-making processes for determining the health services and interventions available to a population that are covered by public funding, also referred to as a “benefit package” or “essential service”. These decision-making processes vary across countries and are discussed across a range of literature on priority setting, including health technology assessment (HTA) and health benefit package design for UHC. Literature on the former (HTA) tends to be concentrated in high-income countries (HICs), however examples in low- and middle-income countries (LMICs) are emerging [8]. Defining a health benefit package (variously termed as essential benefit package, priority benefit package, or health service package) is identified as a key strategy for attaining UHC [9-12], and this literature tends to focus on LMICs. Benefit package design is a priority setting process of determining the package of services and technologies available to a population that are funded by public budgets [11].

Setting priorities for UHC has been characterised as an inherently political and value-laden process, which requires not only technical inputs but also deliberation among stakeholders with diverging values and interests [11,13,14]. Indeed, the WHO technical working group on benefit packages has enshrined this in one of their key principles: “Essential benefit package design should be democratic and inclusive with public involvement, also from disadvantaged populations” [15].

Abelson et al. (2016) identify four broad rationales for CSP, categorised as serving democratic, methodological, instrumental, and developmental goals [16]. We use these categories here to summarise the diverse rationales found in the wider literature. Democratic goals include improving the legitimacy and fairness of decision-making [5,8,17-20], including through promoting accountability [5] and transparency [16]. Here, engagement is intended to improve the representation of [6,17,19] and responsiveness to the primary beneficiaries of health systems [5,21], which is especially crucial in the context of public, tax-financed health services [16]. Participatory approaches may also help to ensure public support and acceptability of decisions [5,10,16,21,22], manage public expectations [19] and promote public ownership, trust, and mitigate risk of legal action [18]. Methodologically, incorporating patient perspectives and social values is intended to provide a more comprehensive approach than only assessing clinical and cost-effectiveness data [14,16,23,24]. Experiential evidence, grounded in the sociopolitical context, is perceived to permit a more accurate assessment of technologies [21,23]. Instrumentally, engagement is understood to improve the quality of decision-making processes [16,18]. This includes their public relevance, sustainability, and contextual validity [8,17,18,21]. It is also expected to improve the likelihood of successful policy implementation [8,19,21,23], including by bringing in stakeholders’ local knowledge about feasibility and potential barriers [17]. Finally, developmental rationales for public engagement include increasing the public’s understanding of health technologies and HTA [16] and of complex policy processes and challenges [19] as well as strengthening their capacity to contribute [16]. Ultimately, engagement can contribute to raising awareness of public entitlements [18], and citizen empowerment more broadly [19].

Given the many rationales for CSP, and the need for further guidance, this review aimed to synthesise existing evidence in the literature on CSP. While the literature examining public participation in priority setting and HTA is relatively dense, the aim of this review is distinctive in synthesising evidence about CSP in the specific context of priority setting and decision-making for health service coverage.

## METHODS

Systematic reviews provide an essential function in synthesising evidence to support decision-making in health policy [25]. In this case, this review was commissioned by the Eastern Mediterranean Regional Office of the WHO. The research team was joined by a topic expert from this office to promote policy relevance [26]. This included the initial development of the review protocol along with the study authors, which was developed using the PICO format to define the Populations, Interventions, Comparators, and Outcomes of interest [27], as detailed in **Table 1**. These PICO components can be expressed in a single research question: What is the state of evidence on effectiveness of, and facilitators and barriers (O), for community and stakeholder (P) participation in determining health service coverage (I) across different populations, contexts, and mechanisms of engagement (C)? Our sub-questions to tackle this overarching question include: 1) what mechanisms of engagement are identified in the literature on CSP in priority setting and decision-making for health service coverage?; 2) how effective are these mechanisms in involving communities in these processes?; and 3) what are the barriers and facilitators to meaningful CSP? Sub-question 2 involves three components of effectiveness: equity, depth, and stage of engagement, as outlined in the conceptual framework below. The review followed the Preferred Reporting Items for Systematic Reviews and Meta-Analyses (PRISMA) guidelines (Table S1 in the **Online Supplementary Document**) [28].

**Table 1:** PICO components of systematic review

**Population**	community members, citizens, the public, patients / consumer of services, community health workers, and other stakeholders
**Intervention**	processes of community and stakeholder participation priority setting and decision-making processes for health service coverage, including the determination of health benefit packages and health technology assessment
**Comparator**	populations, country contexts, and mechanisms of engagement
**Outcomes**	barriers and facilitators; effectiveness of participation mechanisms (including representative and distributional equity implications, depth, and stage of engagement)

### Search strategy and selection criteria

The research team selected electronic databases and search terms in consultation with an information specialist. Three databases were selected for the review: Web of Science, Ovid Global Health, and PubMed Central. These databases were searched on September 1, 2020, with no limits applied on the type of publication or the country of study. Limits were applied on language (English only) and publication date (2000 onwards). Detailed search strategies for the three databases are outlined in Table S2 in the **Online Supplementary Document**.

Citations for all retrieved studies from each of the databases were imported into a reference manager [29] and all records were manually de-duplicated. The remaining records were imported into Rayyan [30] for title and abstract screening by two analysts (MA and RS). Inclusion and exclusion criteria, identified in **Table 2**, were applied in this screening, and further refined following pilot screening and through check-in calls during the review, in consultation with the lead researcher / principal investigator (AK). We excluded review studies; however, these have informed our study and are incorporated in the introduction and discussion sections.

**Table 2:** Inclusion and exclusion criteria

Inclusion criteria	Exclusion criteria
Population: community-level actors (community, citizens, public, patients, community health workers) and other stakeholders, broadly defined	Population: community actors and stakeholders involved / associated with private health system
Intervention: community and stakeholder engagement in decision-making related to determining health service coverage, including defining health benefit packages and assessment of interventions, technologies, and pharmaceuticals for availability through public, population-wide health services provided by government departments or agencies	Intervention: community and stakeholder engagement in private health systems; needs assessment; review, development, and implementation of clinical guidelines; community engagement in designing health interventions including health education and promotion; research; social accountability and social audits, e.g. at facility or community level; intervention studies piloting an approach to CSP without links to government decision-making processes; studies determining attitudes toward rationing and priority setting; and other forms of priority setting such as individual patient treatment order priority, attitudes to different categories of patients, health governance priority setting, and health goals priority setting
Timeline and geography: 2000 to present, no restriction in geography	Timeline: prior to year 2000
Publication types: peer-reviewed, empirical original articles	Publication types: editorials, commentaries, conference proceedings, grey literature, reviews
Language: English	Language: all languages except English

CSP – community and stakeholder participation

The two analysts double screened all the records and compiled a list of studies included by both, and studies for which there were discrepancies. The full texts of discrepant studies were reviewed and discussed by the research team with reference to relevance criteria. This process led to further refinement of the study scope and discussion of emerging themes. One additional study was identified for inclusion during the data extraction stage from reviewing the reference lists of key studies.

### Quality assessment

Quality criteria were drawn from the systematic review by George et al. [31] which were derived from the Critical Appraisal Skills program and other measures of rigour in health systems research. Each study in our final sample was reviewed for four elements of study quality: sampling, data collection methods, analysis, and trustworthiness (Table S3 in the **Online Supplementary Document**). Most studies included sufficient information about sampling, data collection methods, and analysis, but did not specify measures to ensure validity or reflexivity. While studies varied in quality along the criteria in the assessment tool, no study was found to be consistently of poor quality, and as such none were excluded on this basis.

### Data extraction and synthesis

All the studies that were included in this review used qualitative methods of data collection and analysis. One approach to cope with the large volume and complexity of information resulting from qualitative research is framework synthesis [32-34]. This method, increasing employed in qualitative and mixed-methods systematic review, was designed to inform policy and practice [32] and is useful when research objectives are set in advance, based on the information requirements of decision-makers, and with limited timeframes [35], as was the case for our study. Framework synthesis begins with a deductive approach based on an a priori conceptual framework, though additional themes may be identified inductively during data analysis [32,33].

In the first of the five stages of framework synthesis [33], familiarisation, members of the research team deepened their familiarity with relevant literature on CSP in health system decision-making, advised by the lead researcher (AK). For the second step, framework selection, no existing framework was identified in the literature that met this study’s research objective. A conceptual framework was therefore developed by the lead (AK) based on background literature and inputs from the research team, as has been done in other framework synthesis studies [33].

The framework includes three key components of the effectiveness of CSP: equity, depth of engagement, and stage of engagement, and captured how they were reported in studies included in our review [5,16,24,36]. The principle of equity is critical for effective and sustainable CSP. The broad conceptualisation of “community” often overlooks the substantial inequalities inherent in societies, amplifying certain voices and making others marginal to decision-making processes (e.g. based on class, caste / indigenous, religious, gender minorities). Yet equity considerations are often neglected in the literature examining CSP, with the exception of Mitton et al. and Razavi et al. who in their reviews found only 38% (n = 190) of cases and 25% (n = 24) of studies, respectively, that reported on the participation of vulnerable populations or groups with special needs [5,7]. Our appraisal therefore involved whether studies report on equity considerations, which we examined through two dimensions. Representative equity captures whether study authors report on the degree to which mechanisms of engagement were representative of the general population and to what extent they reached marginalised populations. Distributional equity captures whether study authors report on the distributional effects of CSP in decisions about services and improving health service coverage, in terms of whose needs, preferences, or views are being prioritised / met.

The second component considers the depth of engagement in decision-making processes. Here, we employed a comprehensive five-degree spectrum that identifies a continuum of progressively deeper engagement in decision-making processes, encompassing and expanding upon Rowe and Frewer’s commonly cited typology [6,7,37] from the lowest level of participation restricted to information sharing (Inform), followed by intermediate stages of “consult”, “involve”, and “collaborate”, and leads up to “empower” representing the highest level of engagement [29], with the potential of transforming existing power relations to make governance more responsive to community needs.

Finally, we examine the stage at which engagement occurs, corresponding with the aspects of the decision-making process that engagement is intended to inform. We adapted the WHO guidance [15] that involves the three Ds of Data, Dialogue, and Decision in UHC. The Data stage involves collecting evidence about the burden of disease and the viability of potential interventions. The Dialogue stage involves appraising the evidence, discussing service and intervention options according to the defined criteria. The Decision stage entails the use of legislation and regulation to implement recommended interventions, including appeals processes. We added a fourth stage, Review, indicating the cyclical process of decision making.

During the third and fourth steps of framework synthesis, indexing and charting, we used a data charting form in Excel for structured extraction of textual data from articles. This charting form included the categories of effectiveness from our conceptual framework, alongside categories to capture the type of decision-making activity, country, governance level (national, sub-national, local), population, mechanism(s) of engagement, and barriers, facilitators, and recommendations. We coded types of decision-making activities inductively and identified three types: priority setting and decision-making processes for health service coverage, HTA, and decision-making processes for pharmaceutical coverage specifically. For the population, we adopted an inclusive approach to conceptualising relevant actors, adapting an existing framework. We used Mitton et al.’s categorisation of “multiple publics” into three groups: 1) “individual citizens speaking on their own behalf”, 2) “organized interest groups supposedly speaking on behalf of their membership”, and 3) “the public as patients or consumers of services” [7]. Our review also includes other non-state stakeholders relevant to decision-making for health service coverage, such as health system actors and other experts, therefore we added a fourth category to capture “other stakeholders”.

Data extraction was conducted independently by the two analysts (RS and MA), with each reviewing half of the studies, communicating regularly to discuss uncertainties. Each analyst also conducted data extraction of two of the other’s assigned papers, and one analyst (MA) subsequently reviewed data extraction for all of the included studies, to verify consistency.

In the fifth and final mapping and interpretation stage of the framework synthesis, we reviewed the extracted data and generated aggregative quantitative summaries of some categories, and conducted interpretive synthesis of other categories, such as barriers and facilitators [38]. Preliminary versions of these study outputs were presented to experts from academia and WHO Eastern Mediterranean regional and country offices for discussion and feedback. This framework synthesis and consultative approach aimed to ensure the policy relevance of study findings.

## RESULTS

**Figure 1:**
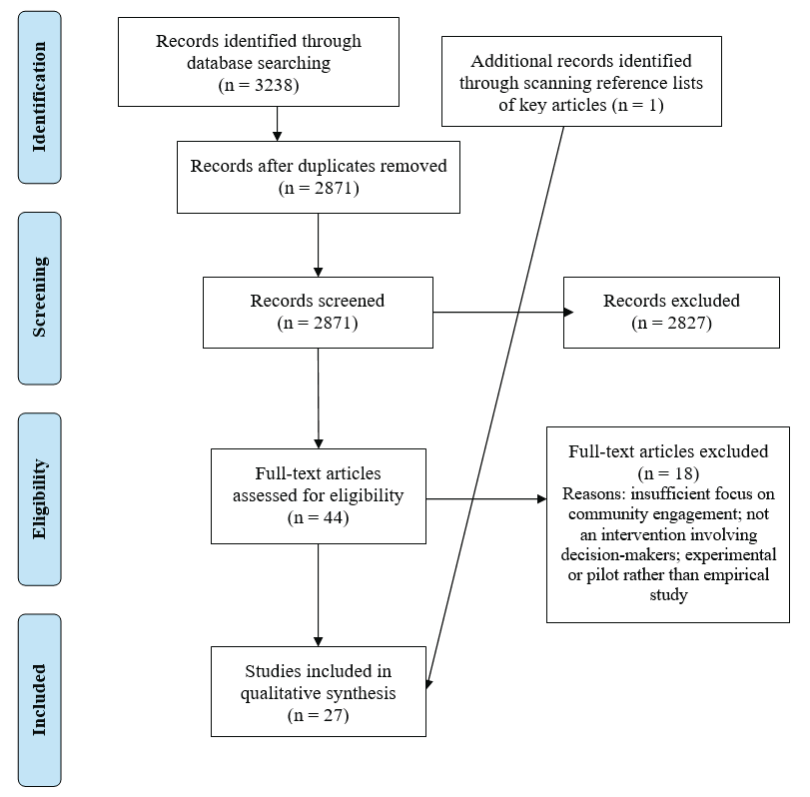
PRISMA search strategy flowchart, adapted from [28].

Our initial search strategy yielded 3238 results, which was reduced to 2871 once duplicates were removed, and 44 following initial title and abstract screening (**Figure 1**). Upon full-text review, a further 18 studies were excluded, and one additional study was identified from scanning the reference lists of articles. The final sample for this review included 27 articles. For the full list see Table S4 in the **Online Supplementary Document**.

While all of the studies included in our review pertained to government-led priority setting and decision-making processes that involved CSP to determine publicly-funded health service coverage, we identified three distinct, yet inter-related, categories of decision-making processes: 1) processes to set priorities and determine health service coverage and packages (12 studies), 2) processes using HTA in particular (11 studies), and 3) decision-making to determine public coverage of pharmaceuticals specifically (four studies). **Table 3** provides an overview of included studies organised according to these three categories. Some studies report findings from multiple countries; each country is reported in our results as a separate case. These cases are from 21 different countries, including 13 high-income, seven middle-income, and one low-income (according to World Bank classification) [39]. Some countries are discussed in multiple studies, including Canada (six studies), the United Kingdom (England and Wales) (UK) (five studies), Thailand (three studies), Brazil (two studies), Kenya (two studies), the Netherlands (two studies), Tanzania (two studies), the United States of America (USA) (two studies), and Zambia (two studies).

**Table 3:** Descriptive overview of included studies

Decision-making activity	Countries’ income level	Health system level*	Engagement mechanisms*
Defining health service coverage and packages: 12 studies (Byskov et al. 2014, Coultas et al. 2018, Danis et al. 2010, Greenberg et al. 2009, Ibe et al. 2017, Kamuzora et al. 2013, Kantamaturapoj et al. 2020, Menon et al. 2007, O’Meara et al. 2011, Razavi et al. 2019, Youngkong et al. 2012, Zulu et al. 2014)	HICs: 4 cases; MICs: 9 cases; LICs: 1 case	National: 4 cases; sub-national: 10 cases; local: 2 cases	Consultation: 12 cases; participation in decision-making committees and boards: 5 cases; participation in district and local planning meetings: 5 cases; appeals: 4 cases
Health technology assessment: 11 studies (Abelson et al. 2007, Cavazza and Jommi 2012, Gagnon et al. 2014, Hashem et al. 2017, Lopes et al. 2016, Menon and Stafinski 2008, Milewa 2006, Rocchi et al. 2015, Silva et al. 2019, Teerawattananon et al. 2016, Yazdizadeh et al. 2016)	HICs: 13 cases; MICs: 3 cases	National: 14 cases; sub-national: 1 case; local: 2 cases	Consultation: 17 cases; participation in decision-making committees and boards: 9 cases; appeals: 4 cases; participation in advisory councils, committees, boards, and panels: 2 cases
Pharmaceutical coverage: 4 studies (Kieslich et al. 2016, Leopold et al. 2020, Regier et al. 2014, Utens et al. 2016)	HICs: 7 cases; MICs: 1 case	National: 9 cases; sub-national: 1 case; local: 1 case	Consultation: 7 cases; participation in advisory councils, committees, boards, and panels: 5 cases

HICs – high-income countries, MICs – middle-income countries, LICs – low-income countries

*Counts reflect multiple health system levels and engagement mechanisms in individual case countries.

The majority of cases focus on decision-making at the national level (27 cases), while 12 examine sub-national levels (such as provinces and districts), and five were undertaken at the local level (including municipality-level and hospital-level decision-making, and clinical commissioning groups). While priority setting for health service coverage usually occurs at the national level, cases at sub-national or local levels are from devolved or decentralised decision-making contexts. Five studies examine multiple levels in a given country, including decision-making at national, sub-national, and / or local levels [13,40-43]. **Table 3** also summarises the range of engagement mechanisms found in studies from each category of decision-making activity.

**Table 4** presents a breakdown of results based on the comparators of engagement mechanisms, country context, and populations involved, and what studies reported about the measures of effectiveness in our conceptual framework including equity considerations, depth, and stage of engagement.

**Table 4:** Quantitative summary of cases corresponding to mechanisms of engagement, country and population comparators, and measures of engagement effectiveness*

			Measures of effective CSP		
**Mechanism**	**Countries**	**Population**	**Equity**	**Depth of engagement^†^**	**Stage(s) of engagement**
Consultations: 34 cases	HICs: 23, MICs: 10, LIC: 1, Australia: 1, Belgium: 1, Brazil: 2, Canada: 6, England and Wales: 5, France: 1, Germany: 1, Iran: 1, Israel: 1, Kenya: 1, the Netherlands: 2, New Zealand: 1, Nigeria: 1, Spain: 1, Sweden: 1, Tanzania: 1, Thailand: 3, Uganda: 1, USA: 2, Zambia: 1	Ind. cit.: 20, int. gr.: 20, patients: 26, other st.: 21	Rep.: 9, dist.: 3	Consult: 34	Data: 27, dialogue: 17, review: 1
Participation in decision-making committees and boards: 14 cases	HICs: 10, MICs: 4, Australia: 1, Brazil: 1, Canada: 2, England and Wales: 4, France: 1, Israel: 1, the Netherlands: 1, Tanzania: 1, Thailand: 2	Ind. cit.: 5, int. gr.: 9, patients: 8, other st.: 10	Rep.: 3, dist.: 1	Consult: 2, involve: 12	Data: 6, dialogue: 12, decision: 10
Appeals: 8 cases	HICs: 4, MICs: 4 Canada: 3, England and Wales: 1, Iran: 1, Tanzania: 1, Thailand: 1, Zambia: 1	Not specified (studies do not report on which population groups submit appeals)	N / A	N / A	Review: 8
Participation in advisory councils, committees, boards, and panels: 7 cases	HICs: 7, Belgium: 1, Canada: 2, England and Wales: 1, New Zealand: 1, South Korea: 1, USA: 1	Ind. cit.: 5, int. gr.: 2, patients: 3, other st.: 3	Rep.: 2, dist.: 1	Consult: 4, involve: 3	Data: 3, dialogue: 5
Participation in district and local planning meetings: 5 cases	MICs: 5, Tanzania: 2, Uganda: 1, Zambia: 2	Ind. cit.: 2, int. gr.: 5, patients: 3, other st.: 3	Rep.: 4, dist.: 2	Consult: 5	Data: 1, dialogue: 5

CSP – community and stakeholder participation, HICs – high-income countries, MICs – middle-income countries, ind. cit. – individual citizens, int. gr. – organised interest groups, other st. – other stakeholders, rep. – representative equity, dist. – distributive equity, N / A – no answer

*Counts represent number of cases, and may reflect multiple mechanisms, countries, population categories, and stages of engagement in some studies.

**^†^**Counts in this column represent the greatest depth of engagement in a particular case, as lesser depths are implied within that (i.e. if a population is consulted then it can be assumed that it has been informed).

### What mechanisms of engagement are identified in the literature on CSP in priority setting and decision-making for health service coverage?

A wide range and number of CSP mechanisms are reported, often with more than one mechanism used in each case. The most common mechanism across both HICs and LMICs is consultation activities (34 cases), including a wide range of in-person and written methods. Examples of these are found in **Box 1** and a detailed list is provided in Table S4 in the **Online Supplementary Document**).

Box 1 **Examples of consultation activities for community and stakeholder participation (CSP)**

• surveys and polling

• workshops

• interactive seminars

• policy roundtable

• citizen laboratory

• citizen dialogue

• citizens’ jury

• public / community forums and debates

• focus groups

• public hearings

• liaising with facility committees

• evidence statements

• “consumer impact” assessments

• written submissions (often to public consultation documents)

• deliberative decision-making simulations

• media and internet-based methods

• soliciting priorities and nomination of technologies and services

See Table S4 in the **Online Supplementary Document** for a detailed overview of mechanisms discussed in each study included in the review.

The second most common engagement mechanism is participation in dialogue and decision-making committees and boards (14 cases), which was identified in both HICs and MICs. These groups are distinct from CSP in councils, committees, boards, and panels that serve an advisory function rather than direct engagement in decision-making. The latter mechanism was identified in seven cases, all in HICs. Participation in district health planning processes, through district- and local-level meetings, was identified in five cases, all in decentralised systems in MICs. Finally, mechanisms for appealing health service coverage decisions are discussed in eight cases, across both HICs and MICs.

Most of these are formal mechanisms occurring within government institutions and structures, however studies were often not clear about whether they were ad hoc and one-off or institutionalised and consistent over time. One factor identified as contributing to the institutionalisation of engagement mechanisms is the existence of legislation and the extent to which the voices of community actors are seen as critical to decision-making in the country’s constitutional and political processes [44,45]. Two notable country examples of legislation to formalise engagement and ensure continuity are found in Thailand and Brazil. Engagement mechanisms in UHC decision-making processes in Thailand, such as annual public hearings and civil society representation on the decision-making board, stem from provisions in the UHC legislation (the National Health Security Act) that provide for inclusion of the voice and concerns of citizens [44]. Similarly, in Brazil social participation is a guiding principle for the Unified Health System enshrined in the country’s constitution and in law, including specifically in legislation pertaining to HTA and decision-making [45].

### How effective are these mechanisms in involving communities in decision-making?

#### Who is engaged and what equity considerations are in place?

Engagement mechanisms identified in this review often involve participants from across the four categories of actors found in our conceptual framework. Examples of “other stakeholders” are found in **Box 2**. These four categories were identified across decision-making activities and mechanisms, mostly evenly distributed. Our findings indicate that patients are slightly more represented within consultation mechanisms, while individual citizens are less represented in decision-making committees and slightly more in advisory councils (**Table 4**).

Box 2 **Examples of other stakeholders identified in community and stakeholder participation (CSP) within studies included in this review**

• government ministry and departmental officials

• political and parliamentary representatives

• health service managers, administrators, and staff

• health care professionals

• health system experts / analysts

• health economists

• academics and researchers

• insurance company representatives

• health technology company representatives

• pharmaceutical company representatives

Significant gaps in the consideration, and / or reporting, of representative and distributional equity in CSP were identified based on our review. Only 12 cases discuss representativeness, with six explicitly reporting on the inclusion of vulnerable, marginalised, or special-needs groups in priority setting processes for health service coverage and a further six discussing the need for diverse representation in CSP [13,40,43,44,46-54]. Twelve cases indicate selection processes and criteria for CSP [13,41,43,44,46,47,49,53,55-58], while no studies identify specific excluded groups and ways to redress this, nor report on the composition of decision-making committees (i.e. according to gender, age, race / ethnicity, sexuality, religion, and other aspects of social position).

Consideration of distributional equity implications of CSP in decision-making about health service coverage is briefly discussed in only five of the 27 studies in this review, and no studies actually assessed or evaluated these [42,43,47,52,57]. For example, in their action research for community engagement in planning processes in Zambia, Zulu et al. assert that the principle of “fairness” in priority setting resonated well with CSP in the study district as it was already a core value in local planning processes. However, fairness was not assessed in relation to the outcomes of the priority setting processes [52]. In Thailand, Youngkong et al. report that equity was a key selection criterion for determining a shortlist of the health interventions to be assessed for inclusion in the UHC benefit package, resulting in the prioritisation of diseases more commonly experienced among those in poverty [57].

#### What is the depth of engagement enabled by CSP mechanisms in decision-making processes?

In terms of depth, most mechanisms identified in this review enable engagement at the level of consultation or involvement. The consultation level entails obtaining public opinions and experiences regarding priorities, values, services, and intervention preferences for health service coverage. Involvement entails working directly with community actors and other stakeholders in decision-making processes. No studies were identified that involve collaborative or empowered engagement, which can be defined respectively as partnering with the public on each aspect of a decision-making process (collaborative), and giving final decision-making authority to community actors and other stakeholders (empowerment).

#### At what stages of decision-making are actors engaged in CSP processes?

Studies included in this review describe engagement at all stages of health service coverage decision-making, though most commonly the data and dialogue stages. Engagement at the data stage includes involvement in nominating and selecting services and interventions for assessment, as well as providing information about patient and public needs and experiences to serve as evidence in assessing options for health service coverage. This was found across income levels and across types of decision-making, including determining health service coverage, HTA, and pharmaceutical coverage.

The dialogue stage usually entails engagement in appraising services and interventions for inclusion in health service coverage. This includes both defining values and selection criteria for appraisal, as well as conducting the appraisal itself by discussing and prioritising among options. Engagement in this stage of decision-making was found across LMICs and HICs, and across types of decision-making. The National Institute for Clinical Excellence (NICE) in the UK is an example of the former, involving patient advocacy groups in determining assessment criteria for HTA [50]. An example of CSP in appraisal exists in Thailand, where multi-criteria decision analysis was used in deliberative processes involving a diverse range of stakeholders in working groups and in the Subcommittee for Development of Benefit Package and Service Delivery, leading to final recommendations for the inclusion of interventions in the UHC benefit package [57].

In ten cases, CSP in decision-making committees and boards entails a role in the decision stage of priority setting for health service coverage. For example, patient representatives’ collaboration in the NICE Appraisal Committee represents a distinctive case in which the committee’s decisions are binding [50,59]. Another example of community engagement in the decision stage was found in Thailand, where five civil society representatives are members of the National Health Security Board, which is responsible for final decisions and implementation for the UHC benefit package [44,57]. In Brazil, a representative of the National Health Council (which represents users of the national health system) participates in the National Committee for Health Technology Incorporation into Unified Health System (Conitec) plenary, from which final decisions serve to advise the Ministry of Health [45]. CSP is similarly found in committees that advise the Ministry of Health in Australia and Israel, as well as in local-level Clinical Commissioning Groups in the UK [53,58,60].

Finally, we identified nine cases of CSP in the review stage, primarily through the establishment of mechanisms for complaints and appeals of health coverage decisions [13,36,44,48-50,61].

### What are the barriers and facilitators to meaningful CSP?

A wide range of barriers and facilitators to CSP were identified in the studies included in our review, though most studies did not include formal evaluation, and many did not report on challenges. We developed a typology to summarise these and conceptualise the different domains of common challenges for engagement, including structural / normative, institutional, procedural, and technical barriers, as presented in Table S5 in the **Online Supplementary Document**. While these can be summarised as such, we have provided references to relevant studies for each element of the table considering the important role of context in shaping barriers and facilitators.

While a range of factors are identified as hindering meaningful engagement, power differentials and divides across different stakeholder groups emerged as a key issue [13,47,50,51,53,55,58,60,62]. This was reflected in a number of studies that reported health administrator resistance to community engagement and the incorporation of evidence based on patient experiences [40,47,50,53,55,63]. It was also reflected in the way certain studies characterised community actors as “lay” participants [50,56-58,60], in contrast to “expert” participants. One study explicitly identified the “well-educated” nature of civil society representatives as a facilitating factor for successful public engagement, without qualifying how engagement is impacted by the education level of community members [44].

In their survey of cancer control decision-makers in Canada, Regier et al. found that their professional and disciplinary background influenced the value they placed on patient-generated evidence and its uptake in decision-making processes. Health care professionals with medical degrees were less likely to use patient input as evidence compared to decision-makers with social science backgrounds. The authors therefore emphasise the use of multi-disciplinary decision-making committees to increase patient and public input in priority setting [40].

## DISCUSSION

Among the 27 studies included in this systematic review, a wide range of mechanisms of engagement, populations, effectiveness outcomes, and barriers and facilitators to meaningful CSP were identified. Studies were concentrated in HICs, which may reflect not only a higher rate of CSP in these countries, but also more formalisation of decision-making processes and institutions for priority setting of health service coverage. Indeed, in a comparative study of four LMICs, Holtorf et al. found that only one had clearly defined HTA processes (Brazil) [8]. As greater formalisation of decision-making is developed in pursuit of UHC [64], existing experiences and recommendations for CSP best practices should be incorporated into country plans.

One of the challenges for clear guidance and identification of best practices to date is the inconsistencies in how CSP is conceptualised and what constitutes meaningful participation. Reporting on who is involved in engagement mechanisms and equity considerations within this is a major gap in the literature; both among studies included in this review as well as the wider literature on participation in health governance. Central to this is how communities are conceptualised and the extent to which studies make a distinction between various stakeholders engaged in decision-making processes. This review revealed a need for further disaggregation of Mitton et al.’s categorisation of three “publics”, including individual citizens, organised interest groups, and individual patients [7]. In particular, the category of “organised interest groups” provides a catch-all phrase capturing a wide variety of groups, from representatives of civil society groups and non-governmental organisations, as well as patient advocacy groups and patient representatives, who may represent quite different perspectives and interests.

Indeed, a number of barriers and challenges for CSP reported in studies relate to equity in the representation of interests within health service coverage decision-making, including de-legitimising patients’ and public views and experience in relation to “experts”. Other studies reported concerns about patient group representatives advocating for the interests of private industry [13,44,55,61,63]. Studies often discussed the engagement of a wide range of stakeholders alongside community actors, without clarifying how they were selected, nor making a distinction between their legitimacy and authority. Indeed, Youngkong et al. state that the presence of multiple players with differing agendas further complicates decision-making processes [20].

The representation of private sector / industry stakeholders in decision-making processes is of particular concern, as the concept of “the public” is “prone to manipulation by those with strong interests” [13]. Due to differences in resources and “powers of persuasion”, the involvement of stakeholders representing professionals and industry interests alongside public actors gives greater voice to the former unless measures are taken to mitigate these imbalances [13]. This echoes the wider literature on priority setting for UHC [12] in calling for the need to consider power differentials in the direct representation of industry actors with vested interests, compared to citizens and patients. To overcome this barrier to effective, equitable CSP, this review highlights the importance of transparent processes and careful consideration of interests when identifying and selecting community members for inclusion in engagement processes.

The distributional equity implications of conflicting interests are evidenced in a study by Yassoub et al. about stakeholders’ views on a health benefit package in Lebanon. They found that the weakest support for improving equity and access to health services came from the private health sector, which unanimously opposed the absence of out-of-pocket payments in the package [65]. A study from Iran further found that private sector and health insurance representatives did not accept the central stewardship of HTA by the Ministry of Health [36]. Studies are deficient in reporting the distributional trade-offs in decision-making and how these are managed. An important challenge for designing and implementing effective community engagement will be to determine how health service decisions will be made in situations of conflicting interests between powerful stakeholders.

One strategy to mitigate conflicts of interests (among others) in order to facilitate effective CSP may be to increase the representation of community-based actors, including particularly marginalised and vulnerable populations. Our study found limited discussion of equity considerations in articles included in our review, and even less successful examples of equitable inclusion in practice. Indeed, the characterisations of “lay” participants and comments about participants’ education levels seem to demonstrate a biased perception of the “right” kind of community actors for participation, and potential inequities and failures in reaching out to the “left-behind” marginalised communities. This suggests at least a failure in tackling, and possibly further contributing to exacerbating, social inequities prevalent in health service recipient communities. We echo other authors in arguing that greater attention needs to be paid to diversity and representativeness within CSP, including key considerations such as clear and transparent nomination and selection criteria for representatives, rationales for the inclusion of particular actors and their role and practices in priority setting and decision-making, and methods to ensure equitable inclusion within participatory spaces to overcome power and resource imbalances [5,19]. These questions should be considered both in planning CSP and in reporting and research about participatory mechanisms.

An important facilitator for effective depth and decisive stage of CSP identified by our study is legislative provisions that compel the institutionalisation and continuity of CSP mechanisms. The examples provided by Thailand and Brazil demonstrate the role played by legal and constitutional provisions in ensuring CSP and promoting its effectiveness. Community engagement in defining the essential package of services for UHC in Thailand is widely recognised as a leading model. The success of engagement mechanisms in this context is understood to stem from the stipulations for engagement and accountability set out in the UHC legislation (the National Health Security Act). This is ultimately the result of civil society advocacy, which led to the inclusion of these provisions through the submission of a draft bill during the drafting of the legislation [44]. In Brazil, health councils and conferences are a fixture of the health system at various levels due to active civil society engagement that has historically contributed to constitutional and legal specifications for social participation in the health system [45]. Both of these country examples demonstrate the contribution of advocacy efforts to achieve rights-based commitments that enable a legacy of participatory governance. Legal requirements also seem to promote the establishment of ongoing engagement mechanisms, ensuring the continuity of CSP. This is significant as ongoing, sustainable methods of public engagement seem to be more effective at securing public engagement in priority setting rather than ad hoc, one-off approaches [7].

In addition to the institutionalisation of engagement mechanisms, the stage at which they occur provides a measure of meaningful CSP. Our findings indicate engagement in all stages of determining health service coverage, however primarily at data and dialogue stages. When engagement did occur in the decision stage, the role of community actors and other stakeholders in contributing to final decision-making was unclear. As one study from Israel noted, the national advisory committee (which includes public representatives) that makes final recommendations on adding health technologies to the national list operates on a consensus basis, therefore the impact of any one committee member is unclear [58].

Across all stages of engagement, evidence of the impacts of CSP on the actual outcomes of decision-making processes for health service coverage was lacking in the literature. Some studies cited anecdotal evidence, including one from Thailand in which the authors note that inputs from annual public hearings led to changes to the organisation and eligibility of services (for example, a limit on the number of birth deliveries included in the UHC service package was eliminated) [44]. The dearth of evidence of impact on decision outcomes may be in part methodological, in that it is often difficult to trace decision-making to a particular input within complex processes. This lack of clarity presents a challenge for generating evidence of engagement effectiveness. It may also present a barrier for CSP, as a lack of feedback from decision-makers on approved priorities reduces the perceived impact of CSP and may discourage engagement [47].

### Limitations

This study was limited by the inclusion of only English language articles. Moreover, while the use of three different databases is comprehensive, it is possible that relevant articles were missed if they were not indexed in one of the selected databases. Grey literature sources were also not included and might have added relevant empirical findings. Our search terms included “health care” or “health services”, which may have missed some relevant HTA studies as that literature may not explicitly mention these terms in titles or abstracts.

Moreover, synthesising qualitative research is challenging, including deciding what to abstract from qualitative studies while navigating the complexity and contextual nature of their findings [66]. We have attempted to mitigate this challenge through the use of the framework synthesis method and a clearly defined conceptual framework to identify the parameters of our analysis. Our results were also limited by the lack of formal evaluation of CSP reported in the literature, and presumably conducted in practice. We echo other reviews and conceptual articles, therefore, in calling for more systematic evaluation of CSP practices [5-7,16].

## CONCLUSIONS

Our systematic review of the literature on CSP in decision-making processes for determining health service coverage found a dearth of relevant studies. Among those that were identified, most were descriptive and provided limited evaluative information. Our assessment based on a conceptual framework of engagement effectiveness identified limited consideration of representative or distributional equity, limited depth of engagement, and a focus on data and dialogue stages of decision-making processes, with some involvement in the decision stage. Overall, our results highlight a need for further research and evidence, for which our conceptual framework offers some key parameters.

This review indicates that while global policy guidelines and resolutions commit to community engagement, such commitment has not translated widely to planning and design processes at country level. Countries that are taking promising steps in this direction seemingly do not pay adequate attention to researching the effectiveness of the mechanisms adopted, and conditions in which CSP may flourish. We call on the international health community to prioritise research in this area to generate evidence that can facilitate the creation of transformative governance and fulfil policy commitments.

Some progress and growing interest in CSP in health system priority setting is reflected in the emergence of new research collaborations and guidance for participation, particularly in the field of HTA [8,14,67-70]. In terms of promoting equitable access to health services globally and more rigorous priority setting approaches, Holtorf et al. discuss a collaborative initiative focused on LMICs in particular, to support cross-country learning and sharing of best practices [8].

Specific to the priority setting and decision-making context of determining health service coverage, our study contributes a first step in systematically reviewing and synthesising evidence of the effectiveness of CSP mechanisms. This may contribute to the inclusion of CSP considerations within technical support for priority setting for UHC, such as that provided by the international Decision Support Initiative (iDSI) [12,14], as well as specific guidance for benefit package design within wider guidance for social participation for UHC [1]. We argue that more attention needs to be paid to representative and distributive equity in engagement practice and research, including specific guidance for selection and meaningful inclusion processes for CSP.

## Additional material


Online Supplementary Document
