# Supplementary material for: Community participation and stakeholder engagement in determining health service coverage: A systematic review and framework synthesis to assess effectiveness
Source: J Glob Health. 2023 May 12;13:04034. doi: 10.7189/jogh.13.04034 (PMC10173679; doi:10.7189/jogh.13.04034)
Supplement: Online Supplementary Document [file jogh-13-04034-s001.pdf]

Supplementary File

Article Title: Systematic review of community participation and stakeholder engagement in determining health service coverage

Journal: Journal of Global Health

Authors' Information:
Megan Arthur
Affiliation: PhD candidate, Global Health Policy Unit, School of Social and Political Science, University of Edinburgh, Edinburgh, UK

Ria Saha
Affiliation: Independent public health researcher, London, United Kingdom

Anuj Kapilashrami (Corresponding author)
Affiliation: Professor, School of Health and Social Care, University of Essex, Colchester, UK, and Founding Chair, Migration Health South Asia Network
Email: a.kapilashrami@essex.ac.uk

Contents

Table S1: PRISMA Checklist (adapted from Moher et al. 2009) 2
Table S2: Search strategy in three databases PMC, Web of Science, and Ovid Global Health (Search date: 1 September 2020) 5
Table S3: Quality assessment of included studies (adapted from George et al., 2015) 6
Table S4: Summary of individual papers included in review 12
Table 5: Barriers, facilitators, and recommendations for CSP 16

Table S1: PRISMA Checklist (adapted from Moher et al. 2009)

Section/topic	#	Checklist item	Reported in section
TITLE			
Title	1	Identify the report as a systematic review, meta-analysis, or both.	Title
ABSTRACT			
Structured summary	2	Provide a structured summary including, as applicable: background; objectives; data sources; study eligibility criteria, participants, and interventions; study appraisal and synthesis methods; results; limitations; conclusions and implications of key findings; systematic review registration number.	Abstract
INTRODUCTION			
Rationale	3	Describe the rationale for the review in the context of what is already known.	Introduction
Objectives	4	Provide an explicit statement of questions being addressed with reference to participants, interventions, comparisons, outcomes, and study design (PICOS).	Introduction
METHODS			
Protocol and registration	5	Indicate if a review protocol exists, if and where it can be accessed (e.g., Web address), and, if available, provide registration information including registration number.	N/A

Eligibility criteria	6	Specify study characteristics (e.g., PICOS, length of follow-up) and report characteristics (e.g., years considered, language, publication status) used as criteria for eligibility, giving rationale.	PICO, Search strategy and selection criteria, Inclusion and Exclusion criteria (Methods section)
Information sources	7	Describe all information sources (e.g., databases with dates of coverage, contact with study authors to identify additional studies) in the search and date last searched.	Search strategy and selection criteria (Methods section)
Search	8	Present full electronic search strategy for at least one database, including any limits used, such that it could be repeated.	Supplementary Table S2
Study selection	9	State the process for selecting studies (i.e., screening, eligibility, included in systematic review, and, if applicable, included in the meta-analysis).	Search strategy and selection criteria; Quality assessment (Methods section)
Data collection process	10	Describe method of data extraction from reports (e.g., piloted forms, independently, in duplicate) and any processes for obtaining and confirming data from investigators.	Data extraction and synthesis (Methods section)
Data items	11	List and define all variables for which data were sought (e.g., PICOS, funding sources) and any assumptions and simplifications made.	Conceptual framework; PICO; Data extraction and synthesis
Risk of bias in individual studies	12	Describe methods used for assessing risk of bias of individual studies (including specification of whether this was done at the study or outcome level), and how this information is to be used in any data synthesis.	Quality assessment (Methods section; Supplemental Table S3)
Summary measures	13	State the principal summary measures (e.g., risk ratio, difference in means).	N/A

Synthesis of results	14	Describe the methods of handling data and combining results of studies, if done, including measures of consistency (e.g., I^2) for each meta-analysis.	Data extraction and synthesis (Methods section)

Table S2: Search strategy in three databases PMC, Web of Science, and Ovid Global Health (Search date: 1 September 2020)

Database	Search string (English only) Timeline 2000- to present (2020)	# of results
PubMed Central (PMC)	((Community Networks[MeSH Terms] OR Community Participation[MeSH Terms] OR Stakeholder Participation[MeSH Terms] OR "community engagement" OR "community participation" OR "community consultation" OR "community involvement" OR "community deliberation" OR "community governance" OR "stakeholder engagement" OR "stakeholder participation" OR "stakeholder consultation" OR "stakeholder involvement" OR "stakeholder deliberation" OR "public participation" OR "public engagement" OR "public consultation" OR "public involvement" OR "public deliberation" OR "citizen participation" OR "citizen engagement" OR "citizen involvement") AND (Health Priorities[MeSH Terms] OR Health Care Rationing[MeSH Terms] OR “service package*” OR “benefit package*” OR “health package*” OR "health technology assessment" OR "priority setting" OR disinvest*) AND (Health Services[MeSH Terms] OR "universal health*"))	1527
Web of Science	TS= ((communit* OR stakeholder* OR public OR citizen) AND (participat* OR engag* OR consult* OR involv* OR deliberat* OR govern*) AND ("Priority Benefit Package" OR "service package*" OR "benefit package*" OR "health package*" OR "health technology assessment*" OR "priority setting" OR "resource allocation" OR disinvest*) AND (healthcare OR "health care" OR "health service*" OR "universal health*"))	1137
Ovid Global Health	((communit* OR stakeholder* OR public OR citizen) AND (participat* OR engag* OR consult* OR involv* OR deliberat* OR govern*) AND ("Priority Benefit Package" OR "service package*" OR "benefit package*" OR "health package*" OR "health technology assessment*" OR "priority setting" OR "resource allocation" OR disinvest*) AND (healthcare OR "health care" OR "health service*" OR "universal health*"))	574

Table S3: Quality assessment of included studies (adapted from George et al., 2015)

Elements of study quality	Articles included in the review (n=27)		
	**Yes**	**Partial**	**No**
	**Sampling**		
**Study area described**	Abelson et al., 2007; Cavazza & Jommi, 2012; Coultas et al., 2019; Danis et al., 2010; Gagnon et al., 2014; Hashem et al., 2018; Ibe et al., 2017; Kamuzora et al., 2013; Kantamaturapoj et al., 2019; Kieslich et al., 2016; Menon et al., 2007; Menon & Stafinski, 2008; Milewa, 2006; O'Meara et al., 2011; Razavi et al., 2019; Regier et al., 2014; Rocchi et al., 2015; Silva et al., 2019; Teerawattananon et al., 2016; Youngkong et al., 2012; Zulu et al., 2014	Greenberg et al., 2009; Lopes et al., 2016; Yazdizadeh et al., 2016	Byskov et al., 2014; Leopold et al., 2020; Utens et al., 2016
**Study area selection explained**	Abelson et al., 2007; Cavazza & Jommi, 2012; Coultas et al., 2019; Danis et al., 2010; Gagnon et al., 2014; Ibe et al., 2017; Kamuzora et al., 2013; Kantamaturapoj et al., 2019; Kieslich et al., 2016; Leopold et al., 2020; Lopes et al., 2016; Menon et al., 2007; Menon & Stafinski, 2008; Milewa, 2006; O'Meara et al., 2011; Razavi et al., 2019; Regier et al., 2014; Rocchi et al, 2015; Silva et al., 2019; Teerawattananon et al., 2016; Youngkong et al., 2012; Zulu et al., 2014	Yazdizadeh et al., 2016	Byskov et al., 2014; Hashem et al., 2018 ; Greenberg et al., 2009, Utens et al., 2016

**Sample criteria mentioned**	Byskov et al., 2014; Cavazza & Jommi, 2012; Coultas et al., 2019; Danis et al., 2010; Gagnon et al., 2014; Hashem et al., 2018; Ibe et al., 2017; Kamuzora et al., 2013 Kieslich et al., 2016; Leopold et al., 2020; Lopes et al., 2016; Menon et al., 2007; Menon & Stafinski, 2008; Milewa, 2006; O'Meara et al., 2011; Razavi et al., 2019; Regier et al., 2014; Silva et al., 2019; Teerawattananon et al., 2016; Utens et al., 2016; YoungKong et al., 2012; Zulu et al., 2014		Abelson et al., 2007; Greenberg et al., 2009; Kantamaturapoj et al., 2019; Rocchi et al., 2015; Yazdizadeh et al., 2016
**Non-participation rates**	Lopes et al., 2016; Regier et al., 2014; Utens et al., 2016		Abelson et al., 2007; Byskov et al., 2014; Cavazza & Jommi, 2012; Coultas et al., 2019; Danis et al., 2010; Gagnon et al., 2014; Greenberg et al., 2009; Hashem et al., 2018; Ibe et al., 2017; Kamuzora et al., 2013; Kantamaturapoj et al., 2019; Kieslich et al., 2016; Leopold et al., 2020; Menon et al., 2007; Menon & Stafinski, 2008; Milewa, 2006; O'Meara et al., 2011; Razavi et al., 2019; Rocchi et al, 2015; Silva et al., 2019; Teerawattananon et al., 2016; Yazdizadeh et al., 2016 Youngkong et al., 2012; Zulu et al., 2014
**Data methods**			
**Data sources listed**	Byskov et al., 2014; Cavazza & Jommi, 2012; Coultas et al., 2019; Danis et al., 2010; Gagnon et al., 2014; Hashem et al., 2018; Ibe et al., 2017; Kamuzora et al., 2013; Kantamaturapoj et al., 2019; Kieslich et al., 2016; Leopold et al., 2020; Lopes et al., 2016; Menon et al., 2007; Menon & Stafinski, 2008; Milewa, 2006; O'Meara et al., 2011; Razavi et al., 2019; Regier et al., 2014;		Abelson et al., 2007; Greenberg et al., 2009

	Rocchi et al., 2015; Silva et al., 2019; Teerawattananon et al., 2016; Utens et al., 2016; Yazdizadeh et al., 2016; Youngkong et al., 2012; Zulu et al., 2014		
**Data collector training/ piloting mentioned**	Danis et al., 2010		Abelson et al., 2007; Byskov et al., 2014; Cavazza & Jommi, 2012; Coultas et al., 2019; Gagnon et al., 2014; Greenberg et al., 2009; Hashem et al., 2018; Ibe et al., 2017; Kamuzora et al., 2013; Kantamaturapoj et al., 2019; Kieslich et al., 2016; Leopold et al., 2020; Lopes et al., 2016; Menon et al., 2007; Menon & Stafinski, 2008; Milewa, 2006; O'Meara et al., 2011; Razavi et al., 2019; Regier et al., 2014; Rocchi et al, 2015; Silva et al., 2019; Teerawattananon et al., 2016; Utens et al., 2016; Yazdizadeh et al., 2016; Youngkong et al., 2012; Zulu et al., 2014
**Data collection described**	Byskov et al., 2014; Cavazza & Jommi, 2012; Coultas et al., 2019; Danis et al., 2010; Gagnon et al., 2014; Hashem et al., 2018; Ibe et al., 2017; Kamuzora et al., 2013; Leopold et al., 2020; Lopes et al., 2016; Menon et al., 2007; Menon & Stafinski, 2008; Milewa, 2006; O'Meara et al., 2011; Razavi et al., 2019; Regier et al., 2014; Rocchi et al, 2015; Teerawattananon et al., 2016; Utens et al., 2016; Yazdizadeh et al., 2016; Youngkong et al., 2012; Zulu et al., 2014		Abelson et al., 2007; Greenberg et al., 2009; Kantamaturapoj et al., 2019; Kieslich et al., 2016; Silva et al., 2019
**Supervision mentioned**	Danis et al., 2010		Abelson et al., 2007; Byskov et al., 2014; Cavazza & Jommi, 2012; Coultas et al., 2019; Gagnon et al., 2014; Greenberg et al., 2009; Hashem et al., 2018; Ibe et al., 2017; Kamuzora et al., 2013; Kantamaturapoj et al., 2019; Kieslich et al., 2016;

			Leopold et al., 2020; Lopes et al., 2016; Menon et al., 2007; Menon & Stafinski, 2008; Milewa, 2006; O'Meara et al., 2011; Razavi et al., 2019; Regier et al., 2014; Rocchi et al, 2015; Silva et al., 2019; Teerawattananon et al., 2016; Utens et al., 2016; Yazdizadeh et al., 2016; Youngkong et al., 2012; Zulu et al., 2014
**Ethics statement mentioned**	Coultas et al., 2019; Ibe et al., 2017; Leopold et al., 2020; Lopes et al., 2016; Menon et al., 2007; Razavi et al., 2019; Regier et al., 2014; Silva et al., 2019; Utens et al., 2016; Yazdizadeh et al., 2016; Zulu et al., 2014		Abelson et al., 2007; Byskov et al., 2014; Cavazza & Jommi, 2012; Danis et al., 2010; Gagnon et al., 2014; Greenberg et al., 2009; Hashem et al., 2018; Kamuzora et al., 2013; Kantamaturapoj et al., 2019; Kieslich et al., 2016; Menon & Stafinski, 2008; Milewa, 2006; O'Meara et al., 2011; Rocchi et al., 2015; Teerawattananon et al., 2016; Youngkong et al., 2012
**Analysis**			
**Methods stated**	Byskov et al., 2014; Cavazza & Jommi, 2012; Coultas et al., 2019; Gagnon et al., 2014; Hashem et al., 2018; Ibe et al., 2017; Kamuzora et al., 2013; Kieslich et al., 2016; Leopold et al., 2020; Lopes et al., 2016; Menon et al., 2007; Menon & Stafinski, 2008; Milewa, 2006; O'Meara et al., 2011; Razavi et al., 2019; Rocchi et al, 2015; Regier et al., 2014; Silva et al., 2019; Teerawattananon et al., 2016; Utens et al., 2016; Yazdizadeh et al., 2016; YoungKong et al., 2012; Zulu et al., 2014		Abelson et al., 2007; Danis et al., 2010; Greenberg et al., 2009; Kantamaturapoj et al., 2019

**Limitations stated**	Cavazza & Jommi, 2012; Gagnon et al., 2014; Lopes et al., 2016; O'Meara et al., 2011; Razavi et al., 2019; Regier et al., 2014; Teerawattananon et al., 2016; Utens et al., 2016; Youngkong et al., 2012; Zulu et al., 2014	Ibe et al., 2017; Kamuzora et al., 2013; Menon & Stafinski, 2008; Yazdizadeh et al., 2016	Abelson et al., 2007; Byskov et al., 2014; Coultas et al., 2019; Danis et al., 2010; Greenberg et al., 2009; Hashem et al., 2018; Kantamaturapoj et al., 2019; Kieslich et al., 2016; Leopold et al., 2020; Menon et al.,2007; Milewa, 2006; Rocchi et al, 2015; Silva et al., 2019
	**Trustworthiness**		
**Triangulation by data source**	Gagnon et al., 2014; Hashem et al., 2018; Kamuzora et al., 2013; Leopold et al., 2020; Zulu et al., 2014		Abelson et al., 2007; Byskov et al., 2014; Cavazza & Jommi, 2012; Coultas et al., 2019; Danis et al., 2010; Greenberg et al., 2009; Ibe et al., 2017; Kantamaturapoj et al., 2019; Kieslich et al., 2016; Lopes et al., 2016; Menon et al.,2007; Menon & Stafinski, 2008; Milewa, 2006; O'Meara et al., 2011; Razavi et al., 2019; Regier et al., 2014; Rocchi et al, 2015; Silva et al., 2019; Teerawattananon et al., 2016; Utens et al., 2016; Yazdizadeh et al., 2016; Youngkong et al., 2012
**Triangulation by respondent**	Kamuzora et al., 2013		Abelson et al., 2007; Byskov et al., 2014; Cavazza & Jommi, 2012; Coultas et al., 2019; Danis et al., 2010; Gagnon et al., 2014; Greenberg et al., 2009; Hashem et al., 2018; Ibe et al., 2017; Kantamaturapoj et al., 2019; Kieslich et al., 2016; Leopold et al., 2020; Lopes et al., 2016; Menon et al., 2007; Menon & Stafinski, 2008; Milewa, 2006; O'Meara et al., 2011; Razavi et al., 2019; Regier et al., 2014; Rocchi et al, 2015; Silva et al., 2019; Teerawattananon et al., 2016; Utens

			et al., 2016; Yazdizadeh et al., 2016; Youngkong et al., 2012; Zulu et al., 2014
**Respondent validation stated**	Cavazza & Jommi, 2012; Gagnon et al., 2014; Rocchi et al, 2015; Teerawattananon et al., 2016; Utens et al., 2016; Yazdizadeh et al., 2016		Abelson et al., 2007; Byskov et al., 2014; Coultas et al., 2019; Danis et al., 2010; Greenberg et al., 2009; Hashem et al., 2018; Ibe et al., 2017; Kamuzora et al., 2013; Kantamaturapoj et al., 2015; Kieslich et al., 2016; Leopold et al., 2020; Lopes et al., 2016; Menon et al., 2007; Menon & Stafinski, 2008; Milewa, 2006; O'Meara et al., 2011; Razavi et al., 2019; Regier et al., 2014; Silva et al., 2019; Youngkong et al., 2012; Zulu et al., 2014
**Reflexivity stated**	Hashem et al., 2018		Abelson et al., 2007; Byskov et al., 2014; Cavazza & Jommi, 2012; Coultas et al., 2019; Danis et al., 2010; Gagnon et al., 2014; Greenberg et al., 2009; Ibe et al., 2017; Kamzoura et al., 2013; Kantamaturapoj et al., 2019; Kieslich et al., 2016; Leopold et al., 2020; Lopes et al., 2016; Menon et al., 2007; Menon & Stafinski, 2008; Milewa, 2006; O'Meara et al., 2011; Razavi et al., 2019; Regier et al., 2014; Rocchi et al, 2015; Silva et al., 2019; Teerawattananon et al., 2016; Utens et al., 2016; Yazdizadeh et al., 2016; Youngkong et al., 2012; Zulu et al., 2014;

Table S4: Summary of individual papers included in review

**Author**	**Number in Reference List**	**Country(ies)**	**Health System Level**	**Mechanism(s)**
Abelson et al., 2007	13	Canada	national and sub-national (provincial)	Participation in advisory councils, committees, boards, and panels<br>Consultation mechanisms: surveys, Citizens' Council, others not specified<br>Appeals mechanism
Byskov et al., 2014	48	Kenya, Tanzania, Zambia	sub-national (district)	Consultation mechanisms: visited villages to identify priorities<br>Participation in district and local planning meetings<br>Appeals mechanism
Cavazza & Jommi, 2012	59	England & Wales, France, Germany, the Netherlands, Spain, Sweden	national	Consultation mechanisms: not specified<br>Participation in decision-making committees and boards
Coultas et al., 2018	60	England & Wales	local (clinical commissioning groups)	Consultation mechanisms: not specified<br>Participation in decision-making committees and boards
Danis et al., 2010	43	USA	local (municipal) and sub-national (state)	Consultation mechanism: Choosing Healthplans All Together (CHAT) deliberative decision-making simulation
Gagnon et al., 2014	63	Canada	local (hospital)	Consultation mechanism: not specified<br>Participation in decision-making committees and boards

Greenberg et al., 2009	58	Israel	national	Consultation mechanisms: nomination of technologies for inclusion<br>Participation in decision-making committees and boards
Hashem et al., 2018	55	England & Wales	national	Consultation mechanisms: written and oral evidence statements<br>Participation in decision-making committees and boards
Ibe et al., 2017	41	Nigeria	national and sub-national (state)	Consultation mechanisms: engaging/ liaising with facility health committees, quarterly seminars/interactive sessions
Kamuzora et al., 2013	47	Tanzania	sub-national (district)	Participation in decision-making committees and boards<br>Participation in district and local planning meetings
Kantamaturapoj et al., 2020	44	Thailand	national	Consultation mechanism: annual public hearings<br>Participation in decision-making committees and boards<br>Appeals mechanism
Kieslich et al., 2016	42	Brazil, England & Wales, South Korea, USA	national (all) and local (England & Wales)	Participation in advisory councils, committees, boards, and panels<br>Consultation mechanisms: online written submissions (Brazil), policy roundtable (USA), and unspecified mechanisms (England & Wales)
Leopold et al., 2020	71	Belgium, New Zealand	national	Participation in advisory councils, committees, boards, and panels<br>Consultation mechanisms: survey, citizen lab, citizen dialogue (Belgium); public forum, face-to-face meeting, written submissions, public debates, public stakeholder event, face-to-face meetings, invited written comments (New Zealand)

Lopes et al., 2016	53	Australia	national	Consultation mechanisms: online, 'consumer impact' assessments<br>Participation in decision-making committees and boards
Menon et al., 2007	49	Canada	sub-national (regional health authorities and provincial boards)	Consultation mechanism: surveys, focus groups, meetings with elected officials, community fora<br>Appeals mechanism
Menon & Stafinski, 2008	46	Canada	local (city-level health region)	Consultation mechanism: citizens’ jury
Milewa, 2006	50	England & Wales	national	Participation in advisory councils, committees, boards, and panels<br>Consultation mechanisms: not specified<br>Participation in decision-making committees and boards<br>Appeals mechanism
O’Meara et al., 2011	62	Kenya	sub-national (district)	Consultation mechanism: soliciting input from facility committees
Razavi et al., 2019	51	Uganda	sub-national (district)	Consultation mechanism: not specified<br>Participation in district and local planning meetings
Regier et al., 2014	40	Canada	national and sub-national (provincial)	Participation in advisory councils, committees, boards, and panels<br>Consultation mechanisms: consultation documents; focus groups; public opinion polling; media and internet-based methods; public hearings

Rocchi et al., 2015	61	Canada	national	Consultation mechanisms: not specified<br>Participation in decision-making committees and boards<br>Appeals mechanism
Silva et al., 2019	45	Brazil	national	Consultation mechanisms: online written submissions, public hearing, invitation to HTA plenary<br>Participation in decision-making committees and boards
Teerawattananon et al., 2016	56	Thailand	national	Consultation mechanism: workshops
Utens et al., 2016	54	the Netherlands	national	Consultation mechanism: incorporating information on patient preferences
Yazdizadeh et al., 2016	36	Iran	national	Consultation mechanism: submissions of topics for HTA<br>Appeals mechanism
Youngkong et al., 2012	57	Thailand	national	Consultation mechanisms: nomination and selection of interventions<br>Participation in decision-making committees and boards
Zulu et al., 2014	52	Zambia	sub-national (district)	Consultation mechanism: submitting lists of priorities<br>Participation in district and local planning meetings

Table 5: Barriers, facilitators, and recommendations for CSP

Category	Barriers	Facilitators/ Recommendations
Structural/ Normative	• power imbalances between institutional actors/decision-makers, private sector, and community[13,47,50,51,53,55, 58,60,62]<br>• structural factors hindering public participation (e.g. gender, poverty)[51]<br>• tokenistic engagement[53,55]<br>• mistrust among actors and in systems involved in CSP and decision-making processes[40,60]<br>• poor alignment or conflicting priorities and interests among stakeholders[13,44,51]<br>• potential biases and co-optation within patient groups by pharmaceutical or other conflicting interests,[13,44,61] or mistrust of patient groups due to perception of this[55,63]<br>• decision-makers' resistance to (and lack of support for) public engagement and rejection of public or patient views and experiences as a source of evidence and input into decision-making[40,47,50,53,55,63]<br>• a perception that the public and patients lack objectivity, or are not sufficiently informed to contribute[40,50]<br>• administrators' fears of loss of control, increasing complexity, and slowing down HTA processes due to CSP[63]<br>• norms of knowledge-generation and evidence-valuation prioritising technical measures or costs over public or patient inputs[40,50,53–55]<br>• lack of inclusiveness in CSP mechanisms[53]<br>• challenge of achieving 'true' representation of community or other relevant group[13,40,45,49,53,57,58,63]	• use external facilitator(s) for CSP[47]<br>• clarify stakeholder groups that should be involved[13]<br>• civil society advocacy to promote participatory norms [41,44,45]<br>• ensure transparency of processes and interests[44]<br>• establish a culture of trust, respect, and openness[61]<br>• implement processes to manage conflicts of interest and balance competing perspectives[61]<br>• foster good, well-established working relationships between community and staff[62]<br>• identify supportive and influential leaders in administrative roles,[42] particularly with previous experience of public involvement, to promote organisational culture norm changes[55]<br>• design CSP to be context-specific and appropriate[59]<br>• consider the use of multi-disciplinary committees including social science backgrounds to foster openness to patient and public input[40]

	● lack of interest and awareness among community actors of the possibilities of participation[48,51,52,63]	
Institutional	● lack of formalised institutional structures, in some cases due to failed or delayed implementation[40,41,51,52,62]<br>● cost and lack of funding for CSP mechanisms[40,47,52,54, 63]<br>● lack of clarity, confusion, and contradictions among decision-makers' understanding of the role of CSP and of public actors and other stakeholders[41,60,62]<br>● Difficulties and contradictions in defining key terms,[54] such as 'communities' and 'representatives'[62], 'stakeholders' and 'the public'[13]<br>● lack of leadership among decision-makers[48]<br>● frequent turnover of institutional staff and the challenge of continuity of institutional knowledge[44,48]<br>● additional staff workload and resources required for CSP [13,54,63]<br>● poor organisational and communication skills and inadequate publicity of engagement opportunities[48,53]<br>● lack of clarity regarding institutional authority or mandate to pursue engagement[40,47,48]<br>● constraints on capacity of decision-makers to accommodate public preferences, undermining public will to participate[44]	● legislative provisions that compel institutionalisation of participation[44,45]<br>● clarify stakeholders' different conceptions of public involvement to foster agreement on processes to be used[53]<br>● ensure clearly defined objectives for CSP[13,54,63]<br>● clear criteria and processes for selection of individuals and representatives for CSP[63]<br>● strengthen leadership and communication skills of managers[48,52,60]<br>● foster political will and identify champions among leadership[55,71]<br>● assign responsibility to reasonably permanent and committed staff[48]<br>● enhance institutional actors' capacity for consultative and inclusive planning processes[52]<br>● provide monetary or non-monetary incentives for community engagement, such as community recognition[41]
Procedural	● lack of agreement on how to enact CSP[62], including the timing of inputs[53]<br>● lack of procedural knowledge among community[47,53]<br>● patient experts feel intimidated in top-down, large, formal, technical meetings[55]<br>● short notice for meetings and inconvenient scheduling[47,53]<br>● lack of preparation among patient experts[55]	● provide orientation for CSP[63]<br>● regularly review and strengthen CSP platforms to ensure effectiveness[44]<br>● establish formal structures for CSP[48]<br>● create pluralist involvement structures[53,55]<br>● increase the number and diversity of patients and stakeholders engaged in CSP[63] and involve them at different stages, particularly earlier[53,71]

	• time constraints on decision-making processes[40,47,53,54, 60,62,63]<br>• processes of engagement are time-consuming and labour-intensive[43,47,63]<br>• ineffective engagement practices[41,53]<br>• unequal opportunities for different participants to speak and participate[50]<br>• lack of feedback from institutional actors and decision-makers on approved priorities, criteria for decisions, and degree to which they reflect community and stakeholders' preferences, reducing perceived impact of participation[47,49,53,55,57,58,63]<br>• participation does not guarantee that community views are fully incorporated in decision-making[58]<br>• insufficient number of representatives on advisory committee[53,55]<br>• limited impact of CSP in outcomes of decision-making [47,52,55,58,60,62]<br>• unclear lines of communication between decision-makers and citizens/patients[41,53]<br>• cost of engagement to community members[43]; lack of financial support for engagement mechanisms (i.e. to reimburse participants for expenses)[47,48,52]<br>• community and other stakeholders' lack of time for engagement[51]	• support patient experts in preparing for meetings[55,63]<br>• develop a standing network of patients and organisations[53] or expert patient committees[45]<br>• establish clear and transparent guidelines and expectations for engagement[13,52,63]<br>• foster community mobilisation by providing tangible returns for the energy they have invested in participation,[62] including clarifying how CSP contributions inform decision-making[13,55] and disseminating decision results[61,63]<br>• design mechanisms that promote active participation[45] and are fun, informative, and accessible to public participants (e.g. Choosing Healthplans All Together (CHAT))[43]<br>• evaluate CSP mechanisms[45] based on targeted evaluation objectives[63]
Technical	• (increasing) complexity and technical nature of decision-making processes[60]<br>• lack of understanding of concepts or evidence involved in deliberations and decision-making,[51,55,57,63] which in one case was identified as being affected by literacy levels[52]<br>• medical and scientific jargon used by HTA committees[63]	• ensure transparency of information and capacity-building[61]<br>• create official documents in clear and consistent formats and language[45,61]<br>• provide training for technical knowledge, participatory processes, priority setting procedures, and complex contexts of decision-making[45,53,55,58,60,61]

	● lack of expertise and understanding about health systems and methods of assessing interventions and priority setting, hindering meaningful CSP[51,53,63]<br>● information and knowledge asymmetries between institutional actors/decision-makers and community,[36,50,53,58,63] and inadequate information sharing[41]<br>● challenge of integrating public and patient inputs with other forms of evidence,[40,54,55,71,71] particularly based on qualitative methods[63]	● leadership from leaders to integrate diverse forms of knowledge,[52,55] including by developing different processes to make use of different forms of evidence[53]<br>● promote patient involvement in research, including through co-production[55]<br>● acknowledge validity of 'alternative' study methods as evidence and clarify guidance on taxonomy and rigour of study designs[54]<br>● improve methodologies for analysing public consultation outputs[45] as well as synthesising and integrating patient preferences with other criteria[54]
